# Recent Advances in Biomedical, Therapeutic and Pharmaceutical Applications of Microbial Surfactants

**DOI:** 10.3390/pharmaceutics13040466

**Published:** 2021-03-30

**Authors:** Chiara Ceresa, Letizia Fracchia, Emanuele Fedeli, Chiara Porta, Ibrahim M. Banat

**Affiliations:** 1 Department of Pharmaceutical Sciences, Università del Piemonte Orientale “A. Avogadro”, 28100 Novara, Italy; chiara.ceresa@uniupo.it (C.C.); emanuele.fedeli@uniupo.it (E.F.); chiara.porta@uniupo.it (C.P.); 2 Center for Translational Research on Autoimmune & Allergic Diseases (CAAD), Università del Piemonte Orientale “A. Avogadro”, 28100 Novara, Italy; 3 Pharmaceutical Science Research Group, Biomedical Science Research Institute, Ulster University, Coleraine, Northern Ireland BT52 1SA, UK; im.banat@ulster.ac.uk

**Keywords:** biosurfactants, antimicrobials, antiadhesive/antibiofilm agents, antiviral activity, wound-healing promoters, immuno-modulation activity, anticancer agents

## Abstract

The spread of antimicrobial-resistant pathogens typically existing in biofilm formation and the recent COVID-19 pandemic, although unrelated phenomena, have demonstrated the urgent need for methods to combat such increasing threats. New avenues of research for natural molecules with desirable properties to alleviate this situation have, therefore, been expanding. Biosurfactants comprise a group of unique and varied amphiphilic molecules of microbial origin capable of interacting with lipidic membranes/components of microorganisms and altering their physicochemical properties. These features have encouraged closer investigations of these microbial metabolites as new pharmaceutics with potential applications in clinical, hygiene and therapeutic fields. Mounting evidence has indicated that biosurfactants have antimicrobial, antibiofilm, antiviral, immunomodulatory and antiproliferative activities that are exploitable in new anticancer treatments and wound healing applications. Some biosurfactants have already been approved for use in clinical, food and environmental fields, while others are currently under investigation and development as antimicrobials or adjuvants to antibiotics for microbial suppression and biofilm eradication strategies. Moreover, due to the COVID-19 pandemic, biosurfactants are now being explored as an alternative to current products or procedures for effective cleaning and handwash formulations, antiviral plastic and fabric surface coating agents for shields and masks. In addition, biosurfactants have shown promise as drug delivery systems and in the medicinal relief of symptoms associated with SARS-CoV-2 acute respiratory distress syndrome.

## 1. Introduction

Biosurfactants (BSs) are a structurally heterogeneous group of biomolecules that share pronounced surface and emulsifying activities. They can be either located on microbial cell surfaces or released in the extracellular space by different bacteria (*Bacillus*, *Lactobacillus*, *Pseudomonas, Burkholderia, Mycobacterium, Rhodococcus, Arthrobacter, Nocardia, Gordonia* and *Acinetobacter*), yeast and filamentous fungi (*Candida, Saccharomyces*, *Starmerella, Trichosporon, Pseudozyma* and *Ustilago*) [1,2]. They are, therefore, mostly classified by their structural features, the producing microorganisms and their molecular mass. BSs have a hydrophilic region (carbohydrate, amino acid, cyclic peptide, phosphate, carboxylic acid or alcohol) and a hydrophobic region (saturated, unsaturated, linear, or branched long-chain fatty acids or hydrocarbon acids). This amphipathic structure allows a reduction in surface tension at the interfaces of phases with dissimilar polarities (liquid–air, liquid–liquid or liquid–solid) [3,4]. They have the ability to form molecular aggregates, including micelles. The micellar aggregation of BSs is originated at the critical micelle concentration (CMC) typically from 1 to 200 mg/L and, interestingly, about 10- to 40-fold lower than that of chemical surfactants [5].

Based on their molecular weight, BSs are commonly divided into two main classes: the low molecular weight compounds efficiently lower surface tension and interfacial tension and are appropriately called “biosurfactants”; conversely, the high molecular weight polymers are more effective as emulsion-stabilizing agents and are usually called “bioemulsifiers”. According to the chemical composition, BSs can be classified into five major groups: glycolipids, lipopeptides, phospholipids, polymeric compounds and neutral lipids [6].

The most widely studied groups of BSs are lipopeptides, such as surfactin, fengycin and iturin, and glycolipids, such as rhamnolipids, sophorolipids, mannosylerythritol lipids and trehalose lipids [7]. Since the 1980s, these amphipathic molecules have been extensively applied in the biodegradation and detoxification of industrial effluents, bioremediation, industrial emulsions and enhanced oil recovery due to their emulsification, wetting, foaming, cleansing, phase separation, surface activity and reduction in heavy liquid viscosity [8,9,10].

BSs might present valuable alternatives to petroleum-based surfactants. Additional advantageous properties, emphasizing the uniqueness of these natural molecules, include the possibility to modify their chemical composition through genetic engineering or the use of biological and biochemical techniques to alter the metabolic end products, thus tailoring them to meet specific functional requirements [11,12]. In addition, they are claimed to be more biodegradable and eco-friendly than synthetic surfactants [13,14,15,16], less toxic and effective even at extremes temperatures, pH conditions, and salinity [6,13,17,18,19].

Despite having a large number of advantages, some disadvantages are also linked to biosurfactants, such as high production cost and the need for purification for some specific applications (e.g., pharmaceutical). Biotechnological processes involved in the synthesis of biosurfactants are rather expensive, and the purification of surfactants is problematic. Several research groups are engaged in finding a solution for cost reductions in biosurfactant production by using easily available and renewable bioresources as cheap raw materials, industrial wastes or by-products [15].

In terms of biodegradability, as water-soluble molecules, BSs may be susceptible to fast biodegradation by other microorganisms, thus limiting hydrocarbon degradation during bioremediation [20]. Additionally, it is also important to remark that for many applications, especially in biomedical and pharmaceutical processes, it would be interesting if biosurfactants were not biodegraded immediately to develop their function in the formulations where they have been included. However, from an environmental point of view, it could represent a problem, not only because of the changes in microbiota caused by the antimicrobial effect of biosurfactants but also due to the costs that could imply their exclusion [21]. Consequently, it is necessary to study the biodegradation process of biosurfactants to establish not only their environmental impact but also to determine their optimal formulation conditions and stability when applied in different industrial sectors [22].

In addition, critical “Life Cycle Assessment”, which typically considers industrial processes from the basic acquisition of raw materials, to the manufacturing of products, consumer use and, finally, the disposal of waste materials. Such approach does not fundamentally show that a biosurfactant has a much lower environmental impact, in terms of greenhouse gas emissions, than petrochemically derived surfactant processes [11].

Studies for potential applications of biosurfactants in the medical field have increased during the past decade; the pertinence in these fields is mostly related to their biological properties, such as their ability to affect cell membrane permeability, emulsification and adhesion to biotic and abiotic surfaces.

**Figure 1:**
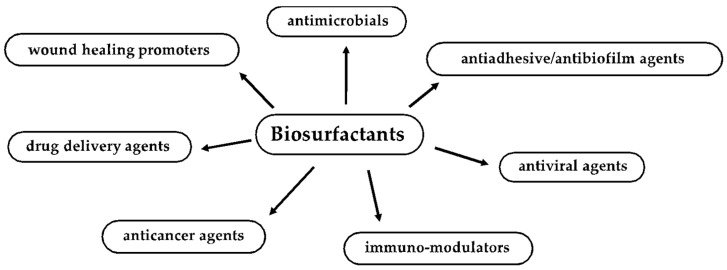
Biomedical, therapeutic and pharmaceutical applications of biosurfactants.

This review focuses on recent advances in the understanding of BSs’ antimicrobial, antiviral, antiadhesive, antibiofilm, wound healing, anticancer and immune-modulatory activities and their promising application in the field of human health [18,23,24,25] (Figure 1). Some critical issues related to the production and application of these molecules will also be presented.

## 2. Biosurfactant Properties and Biological Activities Useful for Biomedical and Pharmaceutical Applications

In nature, BSs modulate various biological activities, including microbial metabolism, motility and survival. These molecules increase the surface areas and bioavailability of hydrophobic water-insoluble substrates and are responsible for the removal of heavy metals from the surrounding environment. They also regulate the attachment/detachment of microorganisms to and from surfaces, mobilization, cell surface conditioning, aggregation at interfaces and surfaces on which the interaction takes place. In addition, cellular differentiation, substrate accession and resistance to toxic compounds are all roles attributed to microbial surface-active compounds [26]. Rhamnolipids, for example, play multiple roles in the survival of microorganisms. They are crucial for the preservation of biofilm architecture and are considered as one of the virulence factors in *Pseudomonas* sp. [27,28] and as part of a natural mechanism evolved to improve the uptake of hydrophobic substrates by bacterial cells. However, current evidence confirms that rhamnolipids are part of a mechanism which controls the fundamental elements of microbial existence, such as the stimulation of bacterial motility, formation and disruption of biofilms, virulence and antimicrobial activity [29].

Overall, BSs confer a selective advantage to the producer microorganism; consequently, they exert antimicrobial activity against other microorganisms that do not produce BSs. BSs can act as virulence factors and as quorum-sensing molecules, regulating the expression of other virulence factors, such as those promoting biofilm formation, maintenance and, ultimately, biofilm dispersal. In addition, they are crucial in maintaining channels for gas and nutrient exchange across, and diffusion into, the biofilm surface and structure [26,27,30,31,32].

**Figure 2:**
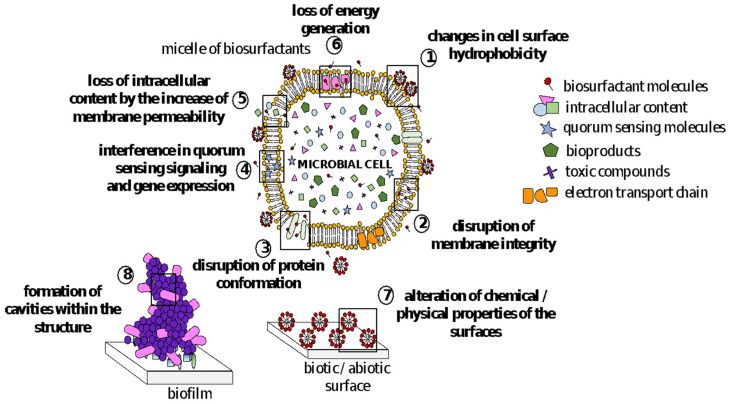
Mechanisms of action of biosurfactants against microbial cell membranes and biofilms.

In recent years, a growing number of studies have pointed out that BSs harbor many biological properties exploitable by biomedical and pharmaceutical fields. BSs mechanism of action on microbial cell surfaces involves binding/attachments to membranes, causing changes in wettability and surface energy, leading to a reduction in hydrophobicity and an increase in permeability through the release of LPS and the formation of transmembrane pores. They, therefore, disrupt membrane integrity, leading to cell lysis and metabolite leakage; loss of membrane functions, such as transport and energy generation processes; and disruption of protein structures (Figure 2) [7,33,34]. Several reports have suggested that, in addition to their direct action against pathogens, biosurfactants are able to interfere with biofilm formation, modulating microbial interaction with interfaces [26] due to changes in surface tension and bacterial cell-wall charge [35].

It is envisaged that more in-depth studies of the natural role of BSs in microbial competitive interactions, cell-to-cell communication, pathogenesis, motility and biofilm formation and maintenance will improve and suggest many other interesting potential applications [5].

## 3. Antimicrobial Activity of BSs

The widespread use of antimicrobials has led to the rapid appearance of an increasing number of drug-resistant microbial strains generating many concerns for future healthcare systems worldwide. According to WHO, antibiotic resistance causes about 700,000 deaths/year, and in Europe alone, about 25,000 deaths/year with an impact cost of about EUR 1.5 billion [36]. In the United States alone, infections due to these types of microorganisms cause 23,000 deaths/year that result in an impact cost of USD 55–70 billion [37].

In this context, microbial metabolites are among the major sources of bioactive compounds. In particular, BSs are very attractive due to their potent antibacterial and antifungal properties for some of them, such as daptomycin [38], and the echinocandins caspofungin [39], micafungin [40] and anidulafungin [41], all of which have already reached a commercial antibiotic status.

### 3.1. Lipopeptides and Glycolipids as Antimicrobial Agents

Lipopeptides and glycolipids are the most commonly reported classes of BSs with antimicrobial activity [42]. In particular, Polymyxin A and Polymyxin B from *Bacillus polymyxa* [43]; surfactin, iturin, fengycin, mycosubtilins and bacillomycins produced by *Bacillus subtilis* [44]; pumilacidin produced by *Bacillus pumilus* [45]; lichenysin from *Bacillus licheniformis* [46]; and viscosin from *Pseudomonas fluorescens* [47] are well known as antimicrobial lipopeptides. Concerning glycolipids, rhamnolipids from *Pseudomonas aeruginosa* [48], sophorolipids from *Candida bombicola* [49] and mannosylerythritol lipids from *Candida antarctica* [50] are the best studied.

Yang et al. [51] discovered a new cationic lipopeptide produced by an environmental strain of *Brevibacillus laterosporus* with marked antimicrobial activities against Gram-positive bacteria, including methicillin-resistant *Staphylococcus aureus* (MRSA), vancomycin-resistant *Lactobacillus plantarum* and *Enterococcus faecalis*, with Minimal Inhibitory Concentration (MIC) values comparable to that of vancomycin.

In 2017, the lipopeptide obtained from *B. subtilis* SPB1, already known for its antimicrobial activity against a wide range of bacteria [52] and phytopathogenic fungi [53], was used as an ingredient in a dentifrice formulation, and its antibacterial activity has been compared to that of a commercial toothpaste.

The BS-based formulation exhibited a remarkable inhibitory activity against *E. faecalis*, *Enterobacter* sp., *Listeria monocytogenes*, *Klebsiella pneumoniae*, *Salmonella enterica*, *Salmonella typhimurium* and *Micrococcus luteus* [54]. Cordeiro et al. [55] observed that the lipopeptide mixture TIM96 was able to kill *Trichosporon inkin* and *Trichosporon asahii* cells within 48 h of co-incubation, via a reduction in cellular ergosterol content and surface hydrophobicity as well as an increase in membrane permeability. Basit et al. [56] isolated 3 strains of *Bacillus cereus* from garden soil whose lipopeptide biosurfactants exhibited significant antibacterial and antifungal activity against *S. aureus*, *Escherichia coli*, *P. aeruginosa*, *K. pneumoniae*, *Aspergillus niger* and *Candida albicans*, with MIC values ranging from 0.52 to 7.6 mg/mL. More recently, Medeot et al. [57] demonstrated that fengycin form *Bacillus amyloliquefaciens* MEP218 was able to induce dramatic alterations in the surface topography of the opportunistic human pathogen *P. aeruginosa* PA01, leading to a decrease in cell height and loss in intracellular content. The surfactin and rhamnolipids mixtures produced by *B. amyloliquefaciens* ST34 and *P. aeruginosa* ST5, respectively, showed a pronounced antimicrobial activity against a broad spectrum of opportunistic and pathogenic microorganisms, including antibiotic-resistant bacterial strains, such as *S. aureus* and *E. coli* and the yeast *C. albicans* [58].

An interesting antimicrobial activity against human pathogens was also reported for the glycolipid obtained by the marine strain *Staphylococcus saprophyticus* SBPS 15 [59]. The biosurfactant completely inhibited the growth of all the tested clinical isolates (e.g., *E. coli, K. pneumoniae, P. aeruginosa, Vibrio cholerae, S. aureus* and *C. albicans*) at concentrations of 4–64 μg/mL. More recently, Valotteau et al. [60] reported the biocidal activity of sophorolipids (SLs)-grafted gold monolayers against both Gram-positive (*E. faecalis*, *Staphylococcus epidermidis* and *Streptococcus pyogenes*) and Gram-negative (*E. coli*, *P. aeruginosa* and *S. typhimurium*) strains. The authors also reported that the exposure of all tested microorganisms to these surfaces caused a significant reduction in cell viability resulting from cell membrane damage. In the same year, Elshikh et al. [61,62] demonstrated the efficacy of mixtures of rhamnolipids and lactonic sophorolipids of different origins in inhibiting the growth of oral bacterial pathogens, finding MIC values against *Streptococcus mutans*, *Streptococcus oralis*, *Actinomyces naeslundii*, *Neisseria mucosa* and *Streptococcus sanguinis* ranging from 0.1 to 0.4 mg/mL. More recently, Sen et al. [63] illustrated the antifungal activity of a rhamnolipid produced by *P. aeruginosa* SS14 against *Trichophyton rubrum*. This study also showed that purified biosurfactant (0.5 mg/mL) effectively induced a loss in cell membrane integrity, suppressed spore germination and hyphal proliferation, altered hyphal morphology in vitro and completely cured induced cutaneous dermatophytosis in 21 days when topically applied to infected mice.

In most studies, the antimicrobial mechanism of action of BS has been ascribed to the well-established disturbing activity on the cell membranes due to the amphiphilic nature of these compounds. However, evidence is emerging of the role of BSs in quorum sensing signaling [29,64,65]. Comparative studies regarding the biosynthesis of rhamnolipids by a strain of *P. aeruginosa* isolated from manure revealed that the cultivation in a selected mixed culture remarkably improved the production of rhamnolipids in terms of maximum yield compared to the axenic culture. This effect was suggested to be associated with interspecies communication via quorum sensing based on AI-2 signaling molecules, demonstrating the significance of interspecies communication for biosurfactant production [66]. This evidence suggests the need to explore the role of BS in microbial competitive interactions.

### 3.2. Biosurfactants from Lactic Acid Bacteria with Antimicrobial Activities

Lactic acid bacteria (LAB) are generally believed to positively influence human health and immune systems. Some of them have shown antimicrobial properties against a broad spectrum of microorganisms, including several pathogens in the intestinal tract and female genital tract due the production of heterogeneous structural biosurfactants [67,68,69]. The biosurfactants produced by *Lactobacillus jensenii* P6A and *Lactobacillus gasseri* P65 showed a marked antimicrobial activity against urogenital tract clinical isolates of *E. coli* (MIC = 16 µg/mL), *Staphylococcus saprophyticus*, *Enterobacter aerogenes* and *K. pneumoniae* (MIC = 128 µg/mL) [70]. In another study, Vecino et al. [71] suggested the use of a glycolipopeptides obtained from a *Lactobacillus pentosus* strain as a “natural” ingredient in cosmetic and personal care formulations due to their efficacy in inhibiting the growth of several microorganisms present in the skin microflora, such as *P. aeruginosa*, *Streptococcus agalactiae*, *S. aureus*, *E. coli*, *S. pyogenes* and *C. albicans*. Most recently, it has also been shown that the biosurfactant from *Pediococcus dextrinicus* SHU1593 is characterized by an interesting dose-dependent inhibitory activity against the planktonic cells of *E. coli*, *E. aerogenes* and *P. aeruginosa*, leading to a complete eradication at 25 mg/mL concentration [72].

## 4. Antiadhesive and Antibiofilm Activity of BSs

Microorganisms exist in their environment as planktonic free living floating cells formation or preferably attached to different surfaces in an immobilized sessile biofilm formation [73,74].

Biofilms are three-dimensional structures in which microbial mono- or multispecies communities (mainly 15–20% of the biofilm volume) with peculiar physiological features are embedded within a self-produced extracellular matrix (80–85%) and separated by a network of open water channels [75,76]. This way of existence provides numerous benefits for microorganisms. Sessile cells interact with one another and communicate by the quorum sensing (QS) system, regulate gene expression and promote all the biological processes and activities useful to their survival within the surrounding environment [77,78]. This leads to an increased ability to withstand environmental stress (e.g., nutrient deprivation, oxygen limitation and pH changes) and to resist the immune system of the host and antimicrobial chemicals [79,80,81,82].

**Figure 3:**
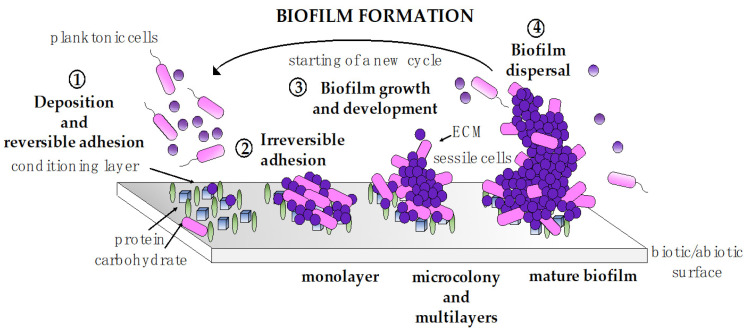
The biofilm lifestyle of microorganisms. ECM: extracellular polymeric matrix.

Biofilm formation is a multistep process that starts with the initial physical attraction of microorganisms to a biotic or abiotic substrate and ends with the dispersion of cells from the biofilm structure [76,83] (Figure 3). It begins when planktonic cells, through bacterial motility and Brownian/Lifshitz–van der Waals forces, settling and reversible adherence to a conditioning substrate (step 1). When the attractive forces (between cells and the surface) counteract the repulsive forces (caused by the negative charge of the cell wall), adhesion becomes irreversible [84]. Cells form a monolayer and start to produce an extracellular polymeric matrix (ECM) (step 2), commonly composed of polysaccharides, proteins, lipids and DNA that will be essential for biofilm structure stabilization and maturation, for nutrient and water recovery as well as for the protection against the surrounding environment [85,86]. Micro-colonies, then, rapidly begin to form, and a biofilm starts to grow as a 3D structure with cells packed in clusters and water channels running between them (step 3) [87]. Finally (step 4), when the waste products begin to accumulate, nutrients become scarce, and the size of the biofilm reaches its maximum volume, and single cells, or small clusters, begin leaving the structure and migrating to new ecological niches and form new biofilms [88].

Biofilms represent a huge scourge in the biomedical field because they are strongly associated with chronic/recalcitrant health care-associated infections (HAI) and antimicrobial resistance [81,89]. Medical device-associated infections are difficult to treat and control and require intensive multidrug therapies and, in most cases, the implant removal as a final solution [90,91]. To date, the search for effective strategies to counteract the formation of biofilms and the onset of resistant microorganisms is a major challenge for the healthcare system [92,93].

In the last 20 years, BSs have proven to be useful in winning this battle, mainly due to their interesting antimicrobial, antiadhesive and antibiofilm properties [18,25]. This is in addition to their propensity to act in synergy with antimicrobials [94,95,96], which in most cases are less effective against biofilms in general and against multispecies biofilms associated with extremely complicated polymicrobial infections.

BSs inhibit biofilms both by decreasing microbial cell viability and reducing microbial adhesion [16,25,68,97,98,99] (Figure 2). The antibiofilm activity of BSs is not only associated with their antimicrobial action by the mechanisms previously described, but it is also related to their ability to form cavities within the biofilm structure [100] and to interfere with quorum sensing signaling and gene expression [101,102]. Furthermore, when applied as coating agents on abiotic surfaces, BSs alter their chemical and physical properties (e.g., reduction in roughness and hydrophobicity) counteracting microbial adhesion [103,104].

The activity of BSs against biofilms on model surfaces, such as polystyrene, glass, silicone and titanium, has already been described in the literature [105,106,107]. Experiments conducted to evaluate the antiadhesive and antibiofilm activities of BSs are commonly carried out in co-incubation or pre-coating conditions. Co-incubation assays are generally utilized for the preliminary assessment of the biological properties of BSs against biofilm formation and to quantify their dislodging effect on pre-formed biofilms. Pre-coating assays are commonly used to evaluate the potential effectiveness of BSs as coating agents in preventing microbial adhesion and, thus, biofilm formation [97].

Examples of recent studies on biosurfactants as antiadhesive/antibiofilm agents and their use in combination with antimicrobials or other natural molecules are reported below.

### 4.1. Lipopeptides and Glycolipids as Antiadhesive/Antibiofilm Agents

Cordeiro et al. [55] observed that the co-incubation of the mixture of surfactin, iturin and fengicin, named TIM96, with *Trichosporon* spp. prevented biofilm formation by inhibiting cell adhesion (up to 96.89%) and caused the dispersal of mature biofilms (up to 99.2%), decreasing their thickness and cell viability. Liu et al. [108] showed that, in co-incubation conditions, surfactin produced by a *B. subtilis* strain strongly affected *S. aureus* adhesion on several materials (glass, polystyrene and stainless steel) and significantly promoted biofilm dislodging. In particular, it was demonstrated that the effect was the result of a decrease in the production of alkali-soluble polysaccharides, the downregulation of *icaA* and *icaD* expression and the alteration of the quorum sensing system by the regulation of the auto inducer 2 activity. In another work, it was also observed that surfactin obtained by *Bacillus safensis* F4, at concentrations of 5 and 10 mg/mL, significantly limited the biofilm formation of *S. epidermidis* S61 with percentages of inhibition of 80–90%, respectively [109]. Giri et al. [110] investigated the antibiofilm potential of lipopeptides produced by *B. subtilis* VSG4 and *B. licheniformis* VS16 against *S. aureus*, *S. typhimurium* and *B. cereus*. The pre-treatment of microtiter plates with biosurfactants considerably inhibited biofilm formation and promoted biofilm eradication with percentages of reduction at the highest concentration tested (5 mg/mL) of 65–82% and 61–76%, respectively.

Regarding the antibiofilm activity of glycolipids, the effects of different types of rhamnolipids and sophorolipids were investigated against some oral bacterial pathogens, such as *S. oralis*, *A. naeslundii*, *N. mucosa* and *S. sanguinis*, by Elshikh et al. [61,62]. BSs significantly inhibited biofilm formation in these strains at a range of 60–90%, in both co-incubation and pre-coating conditions as well as being able to dislodge pre-existing 12-h-old biofilms at a range of 50–100% for all the tested strain.

Recently, Ceresa et al. [111] investigated the coating of silicone elastomer discs with rhamnolipid R89 (composed of mono- (75%) and di-(25%) rhamnolipid families, produced by the clinical isolate *P. aeruginosa* 89). They reported that coated silicone discs reduced both biofilm biomass and metabolic activity (~71% for *S. aureus* and 65% for *S. epidermidis*) up to 72 h, without affecting cell viability and preserving the biocompatibility required for leaching products. In addition, it was shown that the presence of R89 solutions efficiently dispersed *S. aureus* and *S. epidermidis* pre-formed biofilms by up to 93% due to the antimicrobial activity of the rhamnolipid mixture. The antibiofilm activity of three sophorolipid mixtures, SLA (acidic congeners), SL18 (lactonic congeners) and SLV (mixture of both congeners), was evaluated against *S. aureus*, *P. aeruginosa* and *C. albicans* biofilm formation and pre-formed biofilms [112]. In co-incubation conditions, BSs inhibited the formation of microbial biofilms up to 90–95%. The absorption of different concentrations of BSs on silicone strongly limited *S. aureus* and *C. albicans* biofilm formation (up to 72%) in a concentration-dependent manner but was ineffective against *P. aeruginosa*. Furthermore, when used to treat 24-h-old biofilms, all three congener mixtures showed biofilm disruption effects of 70%, 75% and 80% for *S. aureus*, *P. aeruginosa* and *C. albicans*, respectively.

Mannosylerythritol lipids (MELs), from *Pseudozyma aphidis*, were also tested against *S. aureus* biofilm formation and pre-formed biofilms on silicone discs, in co-incubation experiments. The MELs had an interesting ability to inhibit/decrease *S. aureus* biofilm biomass and metabolic activity by their bacteriostatic/bactericidal effect on sessile cells [113]. *Rhodococcus fascians* BD8, isolated from Arctic soil, produced a trehalose lipid with significant antiadhesive properties against *Proteus mirabilis*, *E. coli*, *Enterococcus hirae*, *S. epidermidis*, *E. faecalis*, *Proteus vulgaris* and *C. albicans*. When absorbed on the polystyrene surfaces, BS exhibited a good concentration-dependent antiadhesive activity strongly influenced by the type of microorganism tested. In addition, the trehalose lipid was also able to inhibit the biofilm formation of *E. coli*, *E. faecalis*, *E. hirae* and *C. albicans* on polystyrene and glass in co-incubation conditions and preserved silicone surfaces from microbial colonization when urethral catheters were incubated or coated with it [114].

### 4.2. Biosurfactants from Lactic Acid Bacteria with Antiadhesive/Antibiofilm Properties

The research conducted by Satpute et al. [115,116] assessed the potency of *Lactobacillus acidophilus*-derived biosurfactants as biofilm inhibitors. They first reported on the use of cell-free biosurfactant (CFBS) as a coating-agent for Polydimethylsiloxane (PDMS) surfaces, PDMS-based Microfluidics (MF) channels and silicone catheters while testing for biofilm formation by different bacterial strains. In all these assays, well-formed biofilms were observed for the control surfaces, whereas CFBS-coated samples had no biofilm formation detected up to 48 h using the crystal violet staining and scanning electron microscopy techniques. Afterwards, the pre-treatment of polystyrene surfaces with cell-associated biosurfactants (CABS) efficiently reduced the adhesion of all the tested strains. In particular, ~80% inhibitions were reported for *S. aureus* and *B. subtilis* and 59–65% for *P. aeruginosa*, *P. putida*, *E. coli* and *P. vulgaris*.

As for the other BSs, the antimicrobial and antiadhesive properties of BS produced by Lactobacilli are usually reported to be related to their well-known abilities to interfere with the membrane functions and energy-generating mechanisms to induce cell membrane rupture and reduce cell surface hydrophobicity and microbial adherence to surfaces [35].

Nevertheless, some findings suggest that in addition to BS activities correlated with their amphiphilic nature, their role as signaling molecules and their interaction with the quorum sensing system might be involved in biofilm formation inhibition.

Tahmourespour et al. [117] investigated and reported on the effect of a protein-like BS produced by the strain *Lactobacillus acidophilus* DSM 20079 on the adherence *Streptococcus mutans* on a glass slide, and showed that the BS was able to interfere with the expression level of adherence genes gtfB and gtfC. In particular, real-time RT-PCR demonstrated that the expression of these genes was decreased in the presence of *L. acidophilus*-derived biosurfactant fraction. In addition, it also could make streptococcal chains shorter. In a more recent work, BS isolated from *Pediococcus acidilactici* and *Lactobacillus plantarum* were able to affect the expressions of biofilm-related genes (cidA, icaA, dltB, agrA, sortaseA and sarA) and to interfere with the release of signaling molecules (AI-2) in the quorum sensing systems of *Staphylococcus aureus* CMCC 26003 [65]. In particular, *Pediococcus acidilactici* BS significantly affected the expression of icaA gene and the release of AI-2 signaling molecules, whereas low concentrations of *Lactobacillus plantarum* BS (12.5 mg/mL) significantly reduced the expression of cidA gene. In addition, agrA and sarA gene expression levels were significantly downregulated in the presence of 50 mg/mL of the two different BS.

Giordani et al. [118] evaluated the ability of a BS isolated from *Lactobacillus gasseri* BC9 and BS-enriched liposomes (BS-LP) against biofilms of methicillin-resistant *S. aureus* strains. They reported that free BS prevented biofilm formation and promoted biofilm eradication for five clinically isolates of MRSA strains, a gentamicin-resistant clinical isolate and a sensitive reference strain, in a dose-independent manner and according to the tested *S. aureus* strain. Interestingly, the produced BS-LP exerted higher antibiofilm properties than the BS alone, demonstrating that phospholipid vesicles can act in synergy with BS. These results, in combination with the evidence that lyophilized matrices containing BS-LP quickly dissolved upon contact with exudate and released reconstituted liposomes, suggested a possible application of this delivery nanocarrier for the prevention of skin and soft tissue infections caused by *S. aureus*.

### 4.3. Synergism of BSs with Other Molecules

As mentioned previously, research has now also developed to understand if the potential antimicrobial/antiadhesive/antibiofilm activities of BSs can act in unison with antimicrobials and improve their efficacy. The use of BSs as adjuvants may represent an effective strategy to counteract the infections caused by various antibiotic-resistant microorganisms [26,119]. An advanced atomic force microscopic investigation involving the combined use of tetracycline antibiotics and rhamnolipids or sophorolipid biosurfactants on methicillin-resistant *S. aureus* biofilms dramatically reduced the bacterial coverage on glass surfaces [120]. The treatment with these combinations at sub-MIC tetracycline concentration resulted in swelling and morphological cell damage. This demonstrated that such combinations work jointly to induce cell damage at lower antibiotic concentrations.

Sophorolipids (SL) from *S. bombicola* MTCC 1910 showed antifungal activity and inhibited C. *albicans* biofilm formation and hyphal growth by downregulating the expression of hyphal-specific genes *HWP1*, *ALS1*, *ALS3*, *ECE1* and *SAP4*, as well as eradicating preformed biofilms by reducing the viability of sessile cells. In addition, SL acted synergistically with amphotericin B (AmB) or fluconazole (FLZ) on *C. albicans* biofilm formation and pre-formed biofilms, leading to a reduction in the Biofilm Inhibitory Concentration (BIC)_80_ (AmB: 4-fold; FLZ: 32-fold) and Biofilm Eradicating Concentration (BEC_80_) (AmB: 8-fold; FLZ: more than 8-fold) [102].

A synergistic effect against a clinical isolate of *C. albicans* was also observed when lipopeptide AC7 (AC7BS) was used along with AmB/FLZ [96]. AC7BS alone was not effective in killing the planktonic and sessile cells of *C. albicans*. Nevertheless, both in co-incubation and pre-coating conditions, AC7BS reinforced the efficacy of the two antifungals in inhibiting the fungal growth and biofilm development, resulting in the lowering of the Minimum Inhibitory Concentration (MIC) and Sessile Minimum Inhibitory Concentration (SMIC_50_) values. This was most probably through its ability to increase membrane permeability, facilitating the entry of the antifungal molecules into target cells as well as its antiadhesive activity.

More recently, rhamnolipid (RL)-coated silver (Ag) and iron oxide (Fe_3_O_4_) nanoparticles (NPs) were synthesized and tested for their applicability in the treatment of biofilms formed by *P. aeruginosa* and *S. aureus* [121]. Compared to RL and uncoated NPs, RL-coated NPs demonstrated enhanced antibiofilm activity against both biofilm formation and pre-formed biofilms due to the synergy between the activities of RL molecules. However, the amphiphilic nature of rhamnolipids results in the decreased hydrophobicity of the surfaces, reducing bacterial deposition/adhesion. In addition, rhamnolipid molecules disrupt the predominating electrostatic interactions between the bacterial cells within biofilms and decrease the overall bacterial population by their antibacterial activity.

Marangon et al. [122] reported developing antimicrobial nanoparticles of rhamnolipids and the biopolymer chitosan (C/RL-NPs). The RL addition reduced the size and polydispersity index of chitosan nanoparticles and increased their positive surface charge, stability and the availability of chitosan-free amino groups on the surface that led to a more effective cell envelope disruption and release of rhamnolipid near bacterial cells. The C/RL-NPs nanoparticles were more effective compared to rhamnolipid or chitosan alone against the Gram-positive *S. aureus* and *S. epidermidis* planktonic cells and biofilm formations. These C/RL-NPs nanoparticles were characterized by lower MIC and minimum bactericidal concentration (MBC) values, as well as by the ability to strongly interact with the biofilm matrix and deliver other antimicrobials that diffuse into the biofilm accelerating sessile cells eradication.

It has to be noted that the use of surfactants as potentiators of antibiotic activity has been explored since the late 1960s. In a landmark study by Suling and O’Leary [123], it was demonstrated that distinct classes of synthetic surfactants increased the uptake of different antibiotics into the pathogen cells. However, the efficacy of the surfactants depended on the antibiotic tested and on the target bacterial species. According to these authors, resistance to compounds with properties of cationic surfactants, such as polymyxins, could confer cross-resistance to other surfactants, having these compounds a common target site—the cell membrane—and a common mechanism of resistance. Other criticalities have been observed for the effect of pulmonary surfactant on antimicrobial activity and for the use of exogenous pulmonary surfactant as a drug delivery system for antibiotics in the treatment of respiratory tract infections [124,125,126]. These critical considerations should be taken into account when mixing biosurfactants with antibiotics or antimicrobial nanoparticles.

Recently, the co-occurrence of resistance to heavy metals (e.g., zinc, cadmium) and antibiotics within human bacterial pathogens has been reported. Heavy metals contribute to the promotion of antibiotic resistance through mechanisms of co-resistance and cross-resistance [127]. Due to this evidence, combined with the known toxic effects of some classic drugs, the hypothesis of identifying possible therapeutic strategies based on the use of natural antimicrobial molecules with the same therapeutic efficacy but less adverse effects have emerged [128].

Díaz De Rienzo et al. [129] investigated the antimicrobial activity of combinations of rhamnolipids with caprylic acid and rhamnolipids with sophorolipids against *P. aeruginosa*, *S. aureus* and mixed culture biofilms. Under BioFlux flow through conditions, after 30 min of treatment, the rhamnolipid–caprylic combination caused almost complete dispersion (90%) of all the 48-h-old biofilms. *S. aureus* and mixed biofilms were also efficiently disrupted by the BSs combination. Interestingly, the treatment with BSs was unable to remove *P. aeruginosa* biofilms but was effective in killing the cells within them. These authors also demonstrated that the attachment and biofilm formation of *P. aeruginosa*, *E. coli* and *B. subtilis* on coverslip glasses were greatly inhibited by the combination of sophorolipids and caprylic acid, compared to the results obtained by the treatments with the molecules alone [130]. Lipopeptide AC7BS-coated discs in combination with the quorum sensing molecule farnesol were evaluated for the ability to counteract *C. albicans* biofilms in simulated physiological conditions [131]. Compared to the efficacy of the single compounds, the antibiofilm activity derived from the combination of the two molecules induced an increased inhibition of *C. albicans* adhesion (up to 74%) and biofilm development (up to 93% at 24 h and 60% at 48 h), with relevant reductions in biofilm-covered surface and thickness. The observed synergism was the result of the combination of BS antiadhesive properties and the ability of farnesol to interfere with the yeast-to-mycelium conversion, a fundamental step for biofilm formation.

**Figure 4:**
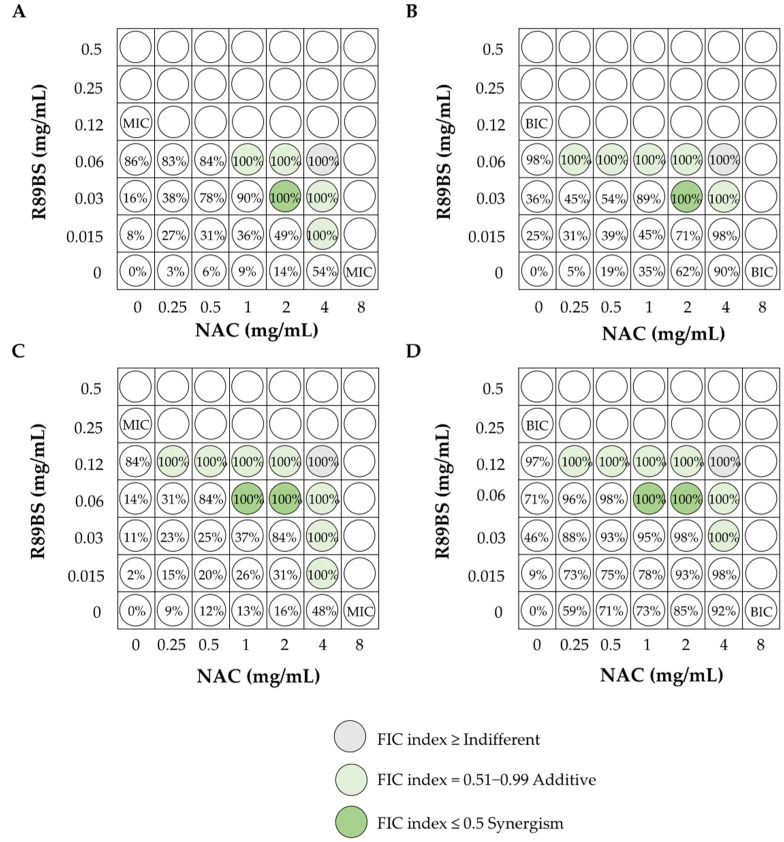
Representation of the results obtained by the Fractional Inhibitory Concentration (FIC) checkerboard assays for the determination of the synergistic activity of R89BS and NAC against the planktonic (**A**,**C**) and sessile cells (**B**,**D**) of the *S. aureus* (**A**,**B**) and *S. epidermidis* (**C**,**D**) clinical isolates. NAC: *N*-acetylcysteine; MIC: Minimum Inhibitory Concentration; BIC: Biofilm Inhibitory Concentration.

Recently, in our laboratory, the antimicrobial and antibiofilm activity of rhamnolipid R89BS (0.015–0.5 mg/mL) in combination with *N*-acetylcysteine (NAC), at a concentration range of 0.25–8 mg/mL, was assessed against *S. aureus* and *S. epidermidis* strains, isolated from central venous catheters, by the checkerboard microtitration method (Figure 4).

The growth inhibition percentage corresponding to each tested condition was calculated, as well as the Fractional Inhibitory Concentration (FIC) index of all sub-MIC/BIC combinations, which induced the complete killing (100%) of bacterial populations. The combined use of R89BS and NAC was effective against planktonic cells (Figure 4A,C) and the biofilm formation (Figure 4B,D) of the tested *S. aureus* (Figure 4A,B) and *S. epidermidis* (Figure 4C,D) strains, and the antibacterial/antibiofilm activity was always greater than that observed for the individual compounds. Furthermore, combinations characterized by an additive or synergistic effect between the two molecules were identified, where MIC/BIC values were halved or decreased by four times at least.

## 5. Antiviral Activity

In the last thirty years, BSs have also been described for their antiviral properties against a variety of enveloped viruses. The inhibitory effects of BSs were the result of the formation of ion channels in viral capsids and lipid envelopes, the loss of proteins involved in viral adsorption/penetration processes and the inhibition of viral membrane fusion [132,133,134]. Naruse et al. [45] were the first researchers that observed the antiviral efficacy of pumilacidins against herpes simplex virus type 1. The mixture of surfactin and fengycin from *B. subtilis* fmbj was found to be able to inactivate Pseudorabies Virus, Porcine Parvovirus, Newcastle Disease Virus and Infectious Bursal Disease Virus in vitro as well as to inhibit the infection and replication processes of Newcastle Disease Virus and Infectious Bursal Disease Virus in porcine kidney and chicken embryo fibroblasts cell lines [135]. More recently, similar results were also described for other lipopeptide mixtures and surfactin analogues against Newcastle Disease Virus and Porcine epidemic diarrhea virus, respectively, corroborating the therapeutic potential of BSs for the development of new antiviral drugs [56,136].

Equally interesting results have also been reported for other classes of BSs. It was demonstrated that the administration of trehalose 6,6′-dimycolate in mice potentiated resistance to influenza virus infection, by inducing the proliferation and lung accumulation of gamma delta TCR^+^ lymphocytes [137]. Sophorolipids exhibited antiviral and sperm-immobilizing activities against human immunodeficiency virus and herpes virus [138,139,140]. Among the tested forms, the diacetate ethyl ester derivatives showed the highest virucidal efficacy against the human immunodeficiency virus type 1, with a reduction in the viral titer of more than 5.2 log units within 2 min [138]. Rhamnolipid PS-17, in free-form and in combination with alginate, was found to be an effective anti-herpes simplex virus agent, by the inhibition of the viral cytopathic effect and the suppression of viral replication in a dose-dependent manner at concentrations lower than the critical micelle concentration of herpes virus [141].

Jin et al. [142] described the ability of rhamnolipids 222B to inactivate two enveloped viruses, bovine coronavirus and herpes simplex virus 1 (HSV-1). They reported that 222B at 0.009% and 0.0045% could inactivate 6 and 4 log PFU/mL of HSV-1 in 5–10 min, respectively and was non cytotoxic at concentration of or below 0.005%. In addition, the authors also explored the possibility to apply rhamnolipids as coating agents on plastic and fabric surfaces for antiviral shields and masks. According to their studies, 50µL of 222B at 0.005% on 1 cm^2^ mask fabrics or plastic surface were able to inactivate ~10^3^ PFU HSV-1 in 3–5 min, paving the way for rhamnolipid coatings on masks to prevent or reduce the spread of enveloped viruses.

The current pandemic outbreak in severe acute respiratory syndrome–coronavirus-2 (SARS-CoV-2) has paved the way for innovative potential pharmaceutical and biomedical applications of BSs. The harmful nature of SARS-CoV-2 is dependent on the integrity of its lipidic envelope, which encloses vital proteins and RNA [143]. The amphiphilic nature of BS allows them to interact directly with the lipid membrane of the coronavirus, breaking up the virus structure and, therefore, diminishing its infectivity [144].

In addition, the propensity of biosurfactants to form micelles structures at their critical micelle concentration (CMC) can also be crucial for application as liposomes for drug delivery to the infection site, preserving its function from the harsh conditions in the body [145]. Applications in pharmaceuticals for gene delivery and the design of molecules to interact with immune system components have also been conceived [146]. Bacterial lipopeptides can act as immunological adjuvants when coupled with antigens. For example, Tripalmitoyl-*S*-glycerylcysteinyl-seryl-serine lipopeptide has been used as an adjuvant by covalent linkage to a synthetic viral peptide that caused the same cytotoxic T cell-mediated immune response observed with a live and infectious virus [147].

BSs use can be also envisaged as a direct treatment for acute respiratory distress syndrome (ARDS), through solubilizing the alveolar substrate and enhancing the clearance of liquid from this region [143]. A recent evaluation of available evidence on the role of biosurfactant in the development of microemulsion drug delivery systems (MDDS) to increase the bioavailability of hydrophobic drugs was carried out by Ohadi et al. [148] who concluded that biosurfactants are an effective biosource for MDDS due to their excellent self-assembling and emulsifying activity properties.

Finally, as natural products, biosurfactants are sustainable compounds with low cytotoxicity, which allows them to be considered for use in handwashes and cleaning agent formulations to prevent the spread of viruses. Taking into account their potential use in such key areas, they are undoubtedly going to be of increasing significance in dealing with the current COVID-19 pandemic [143]. The difficulty with such potential applications is the need for cost-effective mass production. The more likely applications are for uses as sanitizers to surfaces of matter and skin, particularly when chemical alternatives are more aggressive to skin in some people. Reviews examining potential approaches for the future uses of BSs in fighting microbial pathogens, such as the SARS-CoV-2 (COVID19) and others, were recently published [143,146].

It is important to note that although none of the antiviral BSc applications have progressed into clinical testing, interests within the scientific communities remain positive, mainly because pre-clinical investigations indicate their potential for pharmaceutical applications.However, the economical and straightforward production methods, large-scale commercialization and effective integration into industrial processes of BSs are essential requirements for providing transformative and effective solutions for fighting disease outbreaks, such as COVID-19.

## 6. Wound Healing

Wound healing is a complex and strictly regulated process that requires a defined succession of overlapping phases, namely, hemostasis, acute inflammation, resolving inflammation, proliferation and remodeling [149]. After injury, circulating inflammatory cells (mainly neutrophils and monocytes) are recruited to the wound area to defend the host from the pathogens that may enter the body through the disrupted epithelial barrier, as well as to remove damaged cells and necrotic debris. Next, the resolution of inflammation allows the following phases of the healing process: the replacement of dead cells through the proliferation of epithelial cells and fibroblasts (proliferation phase) and the scar tissue formation through the continued deposition and reorganization of extracellular matrix and blood vessels (remodeling phase) [150,151,152]. Failure of one of these phases, due to a dysregulated immune response or insufficient oxygenation, impairs the healing process, leading to important health care burdens: chronic ulcers on one side; fibrosis and permanent scarring on the other side [149,153]. Chronic wounds annually affect 20 million individuals worldwide, which represents a substantial economic burden to healthcare systems, and are often associated with microbial infections that impede the ability of dermal and epidermal cells to respond to reparative stimuli, leading to delayed healing and severe comorbidity and mortality [154,155,156]. In addition, the development of microbial biofilms further complicates an already unfavorable clinical outcome due to their resistance to environmental stresses, drugs and chronic inflammation [157,158,159].

For these reasons, the management and treatment of wounds as well as biofilm prevention is a priority for both clinicians and researchers [160]. In this context, BSs have recently emerged as promising agents capable of promoting wound healing in association with low irritancy and high compatibility with human skin [161,162]. Indeed, BSs are devoid of inflammatory activity while having some antimicrobial and antioxidant properties. In addition, several studies indicate that different BSs promote the proliferation of fibroblasts and epithelial cells and faster collagen deposition, thus leading to an accelerated and improved healing process [163,164,165].

The beneficial effects of surfactin produced by *Bacillus stratosphericus* sp. A15 for wound healing have been demonstrated by Sana et al. [166]. They showed that the lipopeptide harbors antioxidant properties and a remarkable antibacterial activity against *S. aureus* and *E. coli*. In vivo, the BS15 ointment avoids skin irritation, accelerates wound closure and enhances tissue regeneration, as demonstrated by the reconstitution of a thick epidermal layer, with well-differentiated keratinocyte, hair follicles and a higher number of intact cells in the dermis layer. Accordingly, Yan et al. [167] recently reported that surfactin A accelerates wound closure in mice by regulating angiogenesis, inflammatory response and cell migration. They demonstrated that surfactin A enhances the switch of M1 macrophages towards the pro-resolving M2 phenotype and confirmed the beneficial properties of this BS in terms of the regeneration of skin appendages and reduction in scar formation.

The glycolipid BSs of *Bacillus licheniformis* SV1 showed good cytocompatibility and enhanced 3T3/NIH fibroblast cell proliferation in vitro. Accordingly, the application of BS ointment on a skin excision wound in rats promoted re-epithelialization, fibroblast cell proliferation and quicker collagen deposition, thus indicating this product as a potential transdermal substitute to improve skin wound healing [168].

In addition, using the same wound model, it has been reported that an ointment containing rhamnolipid (5 g/L) enhanced wound closure by reducing inflammation and increasing collagen deposition without inducing skin irritation [169].

More recently, More et al. [170] pointed out that BS-containing formulations have improved wound healing properties compared to commercial chemical based ointment. Indeed, in comparison to povidone ointment, the treatment of rat wounds with a sophorolipid-sericine gel induced faster wound contraction, closure and healing in association with enhanced fibroblast proliferation, angiogenesis and keratinization.

Although none of these BSs have advanced to clinic applications, the growing preclinical evidence points to their beneficial activities in the treatment of wounds and supports their pharmaceutical application potential.

In addition to wound healing, BSs could be suitable substitutes for chemical surfactants in current cosmetic and personal skincare pharmaceutical formulations. Indeed, in addition to their antimicrobial and surface moisturizing effects, BSs have lower toxicity and improved skin compatibility than the currently used chemical compounds [171].

In spite of all these promising properties, challenges of very low production yields, difficulty in obtaining pure and standardized products and expensive downstream production processes still represent major limitations for their use in large-scale sustainable pharmaceutic and cosmetic products.

## 7. Anticancer Activity of BSs

According to WHO [172], cancer is the second highest cause of death worldwide. It accounted for 8.8 million deaths in 2015 and has risen over the years since. Despite the development of new promising therapeutic strategies, chemotherapy remains the cornerstone of anticancer treatment. Several drugs used to target cancer cells are based on molecules that are isolated from natural sources, (e.g., plants, microorganisms, vertebrates, and invertebrates) [173] and are endowed with cytotoxic activity for highly proliferative cells. This low specificity towards tumor cells along with the chemo-resistance of many cancer cells represents the Achille’s heal of chemotherapeutic strategies [174,175,176]. Therefore, many efforts aim to identify new anticancer agents that selectively target and sensitize cancer cells to currently used chemotherapeutics [177].

In addition to being new drug candidates in the antimicrobial/antibiofilm field, in recent years, a growing number of studies have indicated BSs as potential antitumor agents [42,178]. As comprehensively reviewed by Fracchia et al. [25], several lipopeptides and glycolipids are capable of inhibiting tumor cell proliferation and survival.

For example, in vitro studies have shown that surfactin exerts anticancer activity against different types of cancer cells, such as Ehrlich ascites, leukemia, breast, colon and liver cancer cells [179]. Surfactin inhibits tumor cell proliferation, viability and migration; however, whether cancer cells might be selectively more susceptible than normal cells is still debatable.

Recently, a glycolipoprotein produced by *Acinetobacter indicus* M6 has shown promising antitumor activity against A549 lung cancer cell lines. In vitro studies have demonstrated an inverse correlation between tumor cell viability and increasing doses (50 to 500 µg/mL) and incubation time (up to 72 h) of the BSs [180]. It is noteworthy that these tested concentrations were nontoxic for normal fibroblast cultures, indicating a selectively higher sensitivity of tumor cells than normal cells. Certainty, in vivo experiments are necessary to validate these in vitro observations.

The overexpression of ATP-binding cassette transporters, such as *P*-glycoprotein (*P*-gp), promotes the efflux of several anticancer drugs leading to multidrug resistance (MDR) [181] and consequently hampering the efficacy of cancer chemotherapy [182]. In this context, different studies have indicated that nanoparticle-based therapeutics could improve drug delivery in solid tumors by coupling an enhanced permeability with an increased retention. Indeed, the small size of nanoparticles allows their passage through the leaky tumor blood vessels, and the impaired lymphatic system prevents their drainage out of the tumor. In addition, nanoparticle-based therapeutics accumulate inside tumor cells by inhibiting or by-passing *P*-gp activity [183,184,185]. The amphiphilic nature of surfactin facilitates its incorporation in nanoformulations (e.g., polymeric nanoparticles and nanofibers, micelles, microemulsions and liposomes), thus enhancing the delivery in the tumor and, consequently, the therapeutic efficacy [179]. In addition to functioning as the active compound, surfactin can be incorporated to improve drug formulation. Accordingly, it has been recently demonstrated that surfactin-based nanoparticles loaded with doxorubicin are able to overcome MDR in human breast cancer cells [186]. In comparison to free doxorubicin, doxorubicin-loaded surfactin (DOX@SUR) nanoparticles showed higher cytotoxicity against different types of human breast cancer cells (MCF-7, T47D and MDA-MD-231 ADR). Mechanistically, DOX@SUR decreased cellular efflux by inhibiting *P*-gp expression. In addition, the uptake of DOX@SUR nanoparticles led to the transportation to lysosomes where the drug is released, allowing its translocation into the nucleus where it exerts cytotoxic activity. Furthermore, in vivo DOX@SUR nanoparticles showed higher accumulation in a murine breast tumor than free doxorubicin, leading to increased antitumor efficacy.

## 8. Immuno-Modulatory Activity of BSs

Interestingly, BS molecules modulate immune responses by affecting the cellular and humoral arms of the immune system [187,188,189]. The immunomodulatory activity of many BSs is primarily exploited by the micro-organism to establish host infection. For example, rhamnolipids support *P. aeruginosa* immune escape by inhibiting the production of antimicrobial peptide (e.g., human beta defensin-2), impairing phagocytic activity and even inducing the lysis of neutrophils and macrophages [190,191,192]. However, the immunosuppressive activities of selected BSs might also be exploited for the treatment of different immune-mediated diseases. In an animal model of sepsis, treatment with sophorolipids protects rats from the lethal effect of septic shock by decreasing the production of nitric oxide (NO) and pro-inflammatory cytokines [193]. In response to sophorolipids, IgE-producing myeloma cells downregulate TLR-2, PAX5 and STAT3, and consequently reduce IL-6 gene expression and IgE production [194]. These results suggest that sophorolipids could mitigate the detrimental effects of IgE-mediated immune responses.

The lipopeptide surfactin produced by *Bacillus* sp. reduces inflammatory response by multiple mechanisms. First, surfactin irreversibly and selectively inhibits phospholipase A2, the enzyme responsible for the release of arachidonic acid from the cell membrane and the subsequent production of crucial inflammatory mediators, such as prostaglandins, leukotrienes and platelet-activating factor [195]. In addition, surfactin limits LPS-induced macrophage activation by hampering MAPK, PI-3 K/Akt and NF-κB activation and by inducing heme oxygenase-1-dependent anti-inflammatory pathways. Overall, these events lead to a reduced expression of inflammatory genes (IFN-γ, IL-6, TNFα, IL-12 and iNOS) and co-stimulatory molecules (CD80, CD40 and MHC II) [196,197,198,199]. Accordingly, pharmaceutical compositions based on lipopeptides or lipoprotein molecules have been patented for treating dysregulated inflammatory diseases [200], and surfactin has been suggested in the prevention of caries and periodontitis due to *P. gingivalis* [198].

In addition to immunosuppressive BSs, selected glycolipid and lipopeptide molecules harbor immunostimulatory effects exploitable for therapeutic interventions, such as vaccines. In this context, the use of BSs as immunological adjuvants is intensively studied.

The cord factor of *Mycobacterium tuberculosis* is a trehalose dimycolate (TDM) able to elicit the activation of innate and adaptive immune response. Although the high toxicity substantially restricts its biomedical use, the trehalolipids produced by the actinobacteria of the *Rhodococcus* genus are emerging as promising immune modulatory molecules with low toxicity [189]. Several studies have indicated that trehalolipids engage macrophage C-type lectin receptors and consequently trigger a signaling cascade that leads to the activation of NF-κB and the expression of pro-inflammatory cytokines [189].

In addition, mycolic glycolipid molecules can be recognized by CD1b molecules that consequently trigger the activation of lymphocytes (e.g., γδ T cells and invariant natural killer T cells) specific for the lipid antigen [201]. Interestingly, glycosphingolipid composition has been recently patented as immune adjuvant enabling human invariant natural killer T cell activation and Th1 cytokine/chemokine production [202]

Different bacterial lipopeptides are being used as nontoxic and nonpyrogenic vaccine adjuvants to enhance host immune response. A considerable improvement of humoral immune response was achieved with the low molecular weight antigens Iturin AL, herbicolin A and microcystin (MLR) coupled to poly-l-lysine (MLR-PLL) in rabbits and chickens [197]. In 2007, Pfizer Products Inc patented some compositions and methods for the formulation of stable adjuvant diluent stock solutions and final adjuvant solutions containing glycolipids, weak acids, alcohols, nonionic surfactants and buffers. More recently, the use of lipopeptides or lipoproteins as an adjuvant in therapeutic or prophylactic vaccinations was patented by Guzman and Muhlradt [203].

Finally, accumulating studies have demonstrated that BSs enhance immune activation and disease resistance in fish, indicating the potential application of BSs in veterinary sciences [188].

## 9. Commercial Applications in the Biomedical and Pharmaceutical Fields

Although many patents have been issued concerning biosurfactant usage for health improvement, real applications in the biomedical and pharmaceutical industries remain quite limited and are summarized, to the best of our knowledge, in Table 1.

As we have seen in this review, a large number of proposals have been introduced for potential commercial applications of biosurfactants in the biomedical and pharmaceutical fields; many of these have not and may not reach any point of significant commercial application in the near future. The key consideration for the exploitation of biosurfactants lies in the functionality of the molecules in the specific formulations required [11]. Following on from functionality is the important consideration of production and downstream processing costs. Some problems must be solved to make the production of BS more profitable and economically feasible by (i) defining protocols to cultivate BS-producing bacterial strains or hyperproducing mutants on renewable cheap substrates; (ii) optimizing growth/production conditions; (iii) implementing large-scale production and recovery processes in order to compete economically with the chemical surfactants [5].

Nevertheless, there is a growing scientific research interest in the improvement of the commercial competitiveness of biosurfactants [146,204]. Among these challenges, biosurfactants are foreseen to impose a significant market share, which is expected to be about USD 5.52 billion by 2022, growing at a compound annual growth rate (CAGR) of 5.6% [205].

## 10. Conclusions and Future Perspective

BSs are emerging surface-active molecules with high potential for a wide range of applications in the biomedical and pharmaceutical fields. BSs are extremely attractive due to their significant antimicrobial (against bacteria, fungi and viruses), antiadhesive and biofilm disruptive properties. Their use, either on their own or in combination with other antimicrobial or chemotherapeutic drugs, might pave the way for a future strategy of prevention and counteraction of microbial infections, biofilm formation and proliferation.

In addition, BSs have recently attracted the attention of the scientific community as a new potential generation of pharmaceutics to be included in anticancer, immunomodulatory, wound healing, cosmetic and drug delivery agents.

However, it should be emphasized that many of these properties can interact and/or affect each other and may results in side effects for different applications, which need to be investigated.

The use of biosurfactants at the commercial level is both timely and essential to reduce the harmful effects of conventional synthetic surfactants on the environment. Challenges associated with the cost-effectiveness of their potential applications and availability, however, remain to be resolved.

## Figures and Tables

**Table 1:** BSs and BS-derived compounds that have reached a commercial status.

Biosurfactant	Function	Application Field
Mupirocin	Antibacterial	Biomedical and pharmaceutical
Oxazolidinone linezolid	Antibacterial	Biomedical and pharmaceutical
Daptomycin	Antibacterial	Biomedical and pharmaceutical
Caspofungin	Antifungal	Biomedical and pharmaceutical
Amphotericin B	Antifungal	Biomedical and pharmaceutical
Micafungin	Antifungal	Biomedical and pharmaceutical
Anidulafungin	Antifungal	Biomedical and pharmaceutical
Rhamnolipids	Emollient, emulsifier	Cosmetic and Personal Skincare
Rapeseed sophorolipids	Antimicrobial, cleansing, deodorant, surfactant	Cosmetic and Personal Skincare
Hydrolyzed palm sophorolipids	Skin conditioning, skin protecting, surfactant	Cosmetic and Personal Skincare
*Madhuca longifolia* sophorolipids	Antioxidant, antiseborrheic, cleansing, emulsifier, surfactant	Cosmetic and Personal Skincare
Sodium surfactin	Cleansing, emulsifying, gel forming, surfactant	Cosmetic and Personal Skincare

## Data Availability

The data presented in this study are available on request from the corresponding author.
